# Factors shaping political priorities for violence against women-mitigation policies in Sri Lanka

**DOI:** 10.1186/s12914-018-0161-7

**Published:** 2018-05-25

**Authors:** Manuela Colombini, Susannah H Mayhew, Ragnhild Lund, Navpreet Singh, Katarina Swahnberg, Jennifer Infanti, Berit Schei, Kumudu Wijewardene

**Affiliations:** 1 Department of Global Health and Development, London School of Hygiene and Tropical Medicine, 15-17 Tavistock Place, London WC1H 9SH, London, UK; 2 Department of Geography, Dragvoll Campus, Building 7, Level 4, Norwegian University of Science and Technology, Trondheim, Norway; 3 AstraZeneca PLC, London, UK; 4 Department of Health and Caring Sciences, Linneaus University, Kalmar, Sweden; 5 Department of Public Health and General Practice, Faculty of Medicine, Norwegian University of Science and Technology, Trondheim, Norway; 6 Department of Community Medicine, Faculty of Medical Sciences, University of Sri Jayewardenepura, Colombo, Sri Lanka

**Keywords:** Violence against women, Gender-based violence, Intimate partner violence, Sri Lanka, Policy analysis, Agenda setting

## Abstract

**Background:**

Although violence against women (VAW) is a global public health issue, its importance as a health issue is often unrecognized in legal and health policy documents. This paper uses Sri Lanka as a case study to explore the factors influencing the national policy response to VAW, particularly by the health sector.

**Methods:**

A document based health policy analysis was conducted to examine current policy responses to VAW in Sri Lanka using the Shiffman and Smith (2007) policy analysis framework.

**Results:**

The findings suggest that the networks and influences of various actors in Sri Lanka, and their ideas used to frame the issue of VAW, have been particularly important in shaping the nature of the policy response to date. The Ministry of Women and Child Affairs led the national response on VAW, but suffered from limited financial and political support. Results also suggest that there was low engagement by the health sector in the initial policy response to VAW in Sri Lanka, which focused primarily on criminal legislation, following global influences. Furthermore, a lack of empirical data on VAW has impeded its promotion as a health policy issue, despite financial support from international organisations enabling an initial health systems response by the Ministry of Health. Until a legal framework was established (2005), the political context provided limited opportunities for VAW to also be construed as a health issue. It was only then that the Ministry of Health got legitimacy to institutionalise VAW services.

**Conclusion:**

Nearly a decade later, a change in government has led to a new national plan on VAW, giving a clear role to the health sector in the fight against VAW. High-level political will, criminalisation of violence, coalesced women’s groups advocating for legislative change, prevalence data, and financial support from influential institutions are all critical elements helping frame violence as a national public health issue.

## Background

Violence against women (VAW) is a global public health and a human rights issue, and represents a major obstacle in overcoming gender inequalities. 1 in 3 women worldwide experience physical and/or sexual violence by a partner or sexual violence by a non-partner [1]. A policy response to addressing VAW is an important step in showing commitment to the issue by government and policy makers. Policy responses to VAW can include legislation enacted by parliament, regulations, strategic plans, guidelines and handbooks, programmes and services, and funding commitments by governments and donors [2]. Many countries worldwide, including in South and South East-Asia, have primarily responded through the adoption of VAW legislation, or revised existing legislation to address VAW [2]. Model frameworks developed by the United Nations (UN) and the Pan American Health Organization (PAHO) emphasize the importance of designing VAW laws and public policies that are multidisciplinary and include the participation of multiple sectors, including health. However, many countries focus on the role of the police and judicial system. A systematic search for VAW legislation identified 124 countries/territories with some type of VAW legislation, of which only 28 mentioned the health sector [2]. This is concerning as it is evident that women experiencing partner violence are more likely to make extensive use of health care services compared to non-abused women [3, 4]. It is now widely recognised that the health sector can play a key role in addressing VAW through early identification, care and referrals to appropriate support services in the community [5]. Recent discussions have also begun to acknowledge the role of the health sector in preventing VAW [5]. Nevertheless, the importance of the health sector in responding to VAW is often unrecognized in legal documents on VAW [6].

### Sri Lankan context

Violence against women is prevalent in Sri Lanka, with intimate partner violence (IPV) (both physical and sexual) being the most perpetrated act [7–9]. A recent scoping review on intimate partner violence in Sri Lanka suggests that IPV prevalence ranges from 20 to 72%, with recent studies suggesting that 25–35% of women have experienced partner violence during their lifetime [10].

Sri Lanka has a fairly well-developed overarching legal and policy framework to combat violence against women, and any forms of discrimination directed towards women, also having ratified several international treaties (e.g. Convention on the Elimination of All Forms of Discrimination against Women, and Convention on the Rights of the Child) which have relevance for VAW. The health sector has also developed a supportive regulatory framework and service response to address violence against women, particularly in recent years. Despite that, the problem of partner violence remained largely underreported by women. A study on men’s attitudes and violence against women in Sri Lanka found that only 32% of females experiencing violence and who sought medical aid had reported the violence and, only 10% had told their families about the violence and the trauma they suffered [9].

Sri Lanka’s strong and multi-level healthcare system offers the opportunity to respond to VAW in a holistic manner, both nationally through policy-making, and with tailored community programmes. However, until very recently, despite evidence on the physical and mental health consequences of VAW, the health sector has not been actively involved in policy responses to VAW in Sri Lanka. Initial national responses to VAW have been focusing on judicial and legal remedies instead, with little focus on health or women’s empowerment strategies.

This paper aims to explore the historical development of the national policy response to VAW in Sri Lanka, explore who the actors involved in the national VAW response were, understand how VAW was framed and what role the health policy sector played in addressing VAW. The paper will focus on one of the most common forms of VAW worldwide, namely intimate partner violence (IPV).

## Methods

This health policy analysis examines current policy responses to VAW in Sri Lanka using the Shiffman and Smith framework [11], which has been used to understand reasons for a limited health-sector policy response. This framework helps explore the actors, ideas, issue characteristics, and political context that have shaped Sri Lanka’s response to VAW.

A literature search was jointly conducted with local partners to identify published evidence and grey literature on policy responses to VAW in Sri Lanka and, specifically, policy responses by the health sector. The following databases were searched: Medline, Global Health, PubMed, Scopus, and Social Policy and Practice, Google, and websites of the Sri Lanka government and relevant ministries, and international donor and aid agencies. The key search terms used to identify documents are the following: gender-based violence, violence against women, intimate partner violence, domestic violence, family violence, health policies, laws, legal and regulatory framework. When not electronically available, identified policies and documents were retrieved by the local partners from the University of Sri Jayewardenepura in Colombo.

Over 50 documents were analysed using a manifest content analysis [12, 13]. Documents analysed included the following: legal documents (3), national policies and plans (29), training manuals and protocols (4), national and donor reports (15), published articles to help understand the context of VAW (9). Textual data was coded systematically and its relevance was checked during this process. Subsequently, the informational content of the policy data was categorized deductively, using pre-conceived themes elaborated at the beginning of the analysis, based on our conceptual framework. These included, among others: key policy actors and their role and influence on VAW; conceptualization of VAW as a national policy issue; enabling policy and regulatory environment around VAW and health; rationale for addressing VAW as a national public health issue; and the rationale for adoption of key health policies around VAW. Direct explanations of some contextual factors and political events were not always possible with the available documentation data, and no interviews with key informants were conducted as part of this study. However, local partners were consulted to help reduce such limitations and enhance our understanding of some data by providing contextual insights.

### Conceptual framework

In order to understand how an issue ultimately gets acted upon by policy-makers, it is essential to understand the first stage of policy-making: agenda setting. Agenda setting is the process by which a particular issue gets onto the policy agenda [14]. Many theoretical frameworks suggest what is required for an issue to reach the policy agenda. Kingdon’s approach proposes the existence of policy streams which need to come together for a window of opportunity to open, leading to an issue reaching the agenda [15]. In 2007, Shiffman and Smith developed a framework of determinants for understanding the political priority of different global health initiatives, used particularly to explain why the Safe Motherhood Initiative was a low priority for global health policy [11]. They proposed four broad determinants influencing whether an issue receives attention (see Table 1 below).

**Table 1:** Shiffman and Smith’s Framework [11]

	Description	Factors shaping political priority
Actor power	The strength of the individuals and organizations concerned with the issue	1. Policy community cohesion: the degree of coalescence among the network of individuals and organizations that are centrally involved with the issue at the global level 2. Leadership: the presence of individuals capable of uniting the policy community and acknowledged as particularly strong champions for the cause 3. Guiding institutions: the effectiveness of organizations or coordinating mechanisms with a mandate to lead the initiative 4. Civil society mobilization: the extent to which grassroots organizations have mobilized to press international and national political authorities to address the issue at the global level
Ideas	The ways in which those involved with the issue understand and portray it	5. Internal frame: the degree to which the policy community agrees on the definition of, causes of, and solutions to the problem 6. External frame: public portrayals of the issue in ways that resonate with external audiences, especially the political leaders who control resources
Issue characteristics	Features of the problem	7. Credible indicators: clear measures that show the severity of the problem and that can be used to monitor progress 8. Severity: size of the burden relative to other problems 9. Effective interventions: the extent to which proposed means of addressing the problem are clearly explained, cost effective, backed by scientific evidence, simple to implement, an inexpensive
Political context	The environments in which actors operate	10. Policy windows: political moments when global conditions align favourably for an issue, presenting opportunities for advocates to influence decision makers 11. Global governance structure: the degree to which norms and institutions operating in a sector provide a platform for effective collaboration

Source: Reprinted from Shiffman and Smith (2007) Copyright 2007

We selected the Shiffman and Smith framework for this study because it provides an approach to examining gaps in political priority. The framework has mostly been used at a global level and we wanted to test its utility for national policy analysis. Specifically, we use it to explore the role of the health sector in the response to VAW in Sri Lanka, and why the health sector has not always been fully active in its involvement. We investigate how the issue of VAW in Sri Lanka has developed by exploring the actors involved; the ways in which VAW has been understood; the severity and prevalence of the issue; and the political context in which VAW has developed.

### Limitations

A policy analysis often includes documents that are informal, such as letters, blogs, and unofficial government documents. Finding these documents proved to be a challenge also for our local research partners, and sometimes led to documents that were of poor quality. In many cases, only small parts of such documents were found to be useful and there was often significant overlap between them. This led to certain documents, which contained more detail, being used more than others within this analysis. Additionally, government websites were sometimes found to be under construction, leading to an inability to access certain documents that may have strengthened the analysis. In searching for documents, limiting the search to English introduced bias, as there were documents, or parts of documents, that were found to be in Tamil and Sinhala. However, most official documents were in English. Lastly, this analysis focused on women in general and did not consider specific groups of women who can be at a higher risk of experiencing VAW, such as those who are displaced or are sex workers.

## Results

A summary of key findings according to Shiffman and Smith’s framework is provided in Table 2.

### Policy actors: What is their role and influence in addressing VAW

The policy-actor landscape around VAW in Sri Lanka has been characterised by an interplay between government, NGOs and individual influences.

**Table 2:** Summarises the key results, according to the study framework

	Description	Factors shaping political priority
Actor power	The strength of the individuals and organizations concerned with the issue	1. Policy community cohesion: Ministry Of Women (MOW) and women’s NGOs allied to pass the Prevention of Domestic Violence Act (PDVA) Bill. However, strong parliamentarian opposition (supporting traditional family values) diluted the Bill. New Government seems more responsive with adoption of VAW Plan in 2016 2. Leadership: the presence of women’s NGOs helped push the PDVA Bill onto the policy agenda. However, cultural and religious views upheld by majority of politicians prevailed and diluted the Act. The presence of a policy champion, internationally recognised for her role as Special Rapporteurs on VAW, helped set VAW high on national agenda. 3. Guiding institutions: MOW was effective in developing national machinery on VAW, though limited recognition and several additional issues to work on. International agencies to influence acceptance of VAW as public health as they funded reports and programmes. 4. Civil society mobilization: grassroots organizations mobilized to press national political authorities to address VAW, though marginal power as not viewed as political actors by the Government.
Ideas	Initially framed as a human right, following international movement. Then, viewed also as a public health issue, though high-level policy-makers viewed it as a threat to family unit	5. Internal frame: All State and on State actors agreed that IPV was a policy issue of concern. However, the politicians and NGO communities did not agree on the definition of IPV, its causes, and solutions to the problem. 6. External frame: President, MPs and Magistrate Court publicly pushed the ideology of family unit, suggesting that IPV is part of married life. Such ideology negatively influenced professionals’ views towards IPV.
Issue characteristics	IPV is pervasive, though no national prevalence data is available (also detailing types of VAW and health consequences). Limited health systems responses.	7. Credible indicators: no clear measures that show the severity of VAW. Data not reliable, primarily coming from cases reported to police. 8. Severity: studies show range from 20 to 72%. However, VAW has to compete with other high mortality non-communicable diseases. 9. Effective interventions*:* Available models of health response for IPV (primarily at hospital level), though anecdotal evidence show their inadequacy.
Political context	Limited political leadership, support and financial resources to IPV. Strong support and influence from international institutions allowed for a health response to VAW.	10. Policy windows: The proclamation of the PDVA Bill was an important political moment, presenting opportunities for women’s advocates to influence decision makers on VAW, and for other Ministries, such as MOH, to legitimise their role in the national response to VAW. Political elections with the regime change also offered a renewed opportunity to push for a concerted national response to VAW. 11. Global governance structure: limited influence from ratifying international VAW governance structures (e.g. Convention on the Elimination of All Forms of Discrimination against Women (CEDAW)), though financial (and technical) influence from international agencies to deliver health systems response.

#### Government institutions and VAW regulatory framework

The Sri Lankan government’s commitment to women’s issues (including VAW) began in the late 1970s with the creation of the Women’s Bureau of the Ministry of Women’s Affairs in 1978 [16]. Since then, additional national mechanisms have been developed to support the country’s international commitments to gender equality (as per ratification of CEDAW), including the elimination of VAW. Among these, there is the Sri Lankan Women’s Charter (1993), which supported the right of women to be free from any form of VAW, including physical, sexual and emotional abuse. It also called for sensitisation of enforcement authorities to raise their awareness on VAW, as well as support to NGOs and community organisations to help provide counselling to women who experienced abuse [17].

‘*The state shall take all measures to prevent the phenomenon of gender-based violence. Such measures shall include provision of support to programmes which provide support and counselling services to women victims of violence*’.

The Women’s Charter also identified ‘health’ as an area which needed to be represented in the National Committee of Women (NCW), the advisory body on women’s issues, created in 1994 to support the implementation of the Charter and monitor the rights protected by the Charter. The NCW was placed under the responsibility of the newly renamed Ministry of Women and Child Affairs (hereafter referred to as Ministry of Women), and assisted in the development of policies and programmes promoting women’s rights and development. VAW became then part of the Ministry of Women’s larger mandate [16]. In 1996, following the Beijing World Conference on Women (1995), the Sri Lankan Government, in collaboration with NGOs, adopted a *National Plan of Action for Women* (NPA) encompassing VAW among its sectors. The first activity related to VAW in the NPA was a review of the legislative framework around discrimination of women under the Penal Code and the establishment of crisis centres for women who experienced abuse [16]. Following the NPA, amendments in the Penal Code (in 1998 and 2006) led to an increase in penalties for rape and the criminalisation of incest, sexual harassment and grave sexual assaults. Though gaps still remain around partner violence and marital rape [18], where cases tend to be settled out of court in Mediation Boards, appointed by the Ministry of Justice [8].

In 2005, the Government of Sri Lanka passed the *Preventive Domestic Violence Bill* (hereafter PDVA Bill), marking the culmination of advocacy campaigns by women’s groups since the 1990s. The Bill aimed to eliminate VAW and led to the recognition of partner violence as a human right violation [19]. This Act incorporated two important concepts from a health perspective: 1) recognises emotional violence before the law and provides counselling services; 2) identifies specific health care providers who are considered suitable to monitor protection orders [20].

Despite the adoption of the PDVA Act and other more recently adopted national social policies and Action Plans containing specific provisions on the elimination of VAW [21, 22], there was still no mention of VAW in high national policies at the time, like the *Ten Year Horizontal Development Plan* (2006–2016), which only incorporates gender related inputs and violence issues around homicides and violence among youth [23].

Despite the presence of these important government political bodies working on VAW, the Ministry of Women’s focus on women has been weakened by the addition of a combination of other issues over time, including development, social welfare and child development. The Women’s Charter, although recognized as the first positive response by the Sri Lankan government to securing the rights of women, did not receive any legislative recognition [16]. Furthermore, the Ministry receives little government funding and carries out much of its work through external donor funding [16], which significantly limits its mandate to create sufficient VAW programming and provide counselling units. Despite these challenges, the Ministry of Women has successfully led the formulation of two important multi-sectoral plans of action to address VAW. The *Plan of Action Supporting the Prevention of Domestic Violence Act* (hereafter Plan of Action 2005) was developed in 2005 [24]. A second plan, the *Policy Framework and National Plan of Action to address Sexual and Gender-based violence* (hereafter referred to as the *National Plan of Action on SGBV) in Sri Lanka*, was recently approved with no reservations in June 2016, and focuses on a concerted effort (with 9 ministries involved) to develop strategies for prevention, intervention and advocacy to address VAW [25].

Besides the Ministry of Women, in the late 1990s, the Ministry of Health (MOH) also started to address VAW in its policies. In 1998, it adopted the *Population and Reproductive Health Policy of Sri Lanka* [26], which identified VAW as an important issue to address being a threat to women’s reproductive health. However, despite this early recognition of VAW by MOH, it was only after the adoption of the PDVA Bill that the MOH started to institutionalise its response to VAW [27]. In particular, the adoption of the Plan of Action in 2005 legitimised the role of the MOH in strengthening existing health and medical services to respond to VAW, and training health providers on how to identify and treat VAW cases [24]. Subsequently, the Ministry of Health developed a *Ten year Health Master Plan 2007–2016* [28], which discussed intimate partner violence and the health needs of women who experience partner violence under the section on maternal health services, specifically recognizing the burden of disease and death caused by partner violence. It also refers to the integration of VAW management into the services of Maternal and Child Health/Family Planning clinics and the provision of adequate services at the level of primary health care (PHC) as some of the important steps in reducing violence [29].

The Family Health Bureau of the MOH’s Directorate of Maternal and Child Health is the national focal point for maternal and child health programmes, and – recently through its Gender and Women’s Health Unit – also oversees all formal VAW training and establishment of VAW services provided through the national hospital system [30]. In 2012, the *Maternal and Child Health (MCH) Policy* was adopted, recognizing the role of the health sector in preventing and responding to VAW, including domestic violence. Clearly detailed in the related MCH Action Plan (under the Gender and Reproductive Health component), its mandate includes capacity building for health staff and calls for the establishment of related health services for abused women [31, 32]. Despite the supportive regulatory health framework for VAW, no separate funds for such activities have been allocated in the state health budget. However, financial support is provided through the allocation of space, staff and infrastructure, while funding for pilot projects and capacity building is provided by international organisations like UNFPA [33].The Ministry of Health’s commitment to address VAW has also being stated in the recently adopted *National Health Strategic Master Plan 2016–2025* [34–36], and its related *National Strategic Framework for Development of Health Services* [37]. Furthermore, its key role in the national multi-sectoral effort to address VAW has also been reiterated in the *National Plan of Action on SGBV* (2016). The related Health Sector Plan of the 2016 *National Plan of Action on SGBV* aims – among other goals – to improve health staff awareness on VAW and to address VAW through reproductive health interventions [25].

#### NGOs

Though governmental agencies such as the Ministry of Women have been playing a key role in the development of the national regulatory machinery against VAW, NGOs have also played a central role in highlighting VAW as an issue needing to be in the public domain. Historically, charitable and faith-based organizations were amongst the first to provide institutionalized services for women who experienced VAW in the country, primarily through referrals to specialized services and providing befriending and legal services [38, 39]. Since after the tsunami in 2004, NGOs have led the way to establishing “Gender-based health desks” at hospitals throughout Sri Lanka, which have a specific mandate to identify and provide support services to women facing violence [16, 40]. Despite being implemented for decades, these services have received scarce political and institutional support from the State. As a result, NGOs have alleged that the government has failed to recognize the health and support needs of abused women in the country [41]. Moreover, the ability of NGOs to continue to provide counselling services for VAW is affected by government officials’ biases towards all NGOs labelling their work as anti-government or pro-separatist [42].

Furthermore, because of their cohesion and networking abilities, NGOs’ convening power has been important for developing advocacy strategies and activities to raise awareness about available laws on VAW, and for challenging social norms amongst the community and policy makers [16]. In particular, women’s NGOs have coalesced to become a particularly active sub-group very influential in legislative reforms to address VAW [39], most notably in drafting the PDVA Bill [17].

#### Experts and international organisations

Similarly, researchers and academics working on VAW have contributed to increasing visibility for VAW, initially focusing primarily on partner violence. VAW became a global concern with the appointment of the first United Nations Special Rapporteur on Violence Against Women from 1994 to 2003. This was equally significant for Sri Lanka because the Rapporteur was the Director of the International Centre for Ethnic Studies (ICES) in Colombo, a leading institution for research and guidance on policies and reforms regarding VAW in Sri Lanka. The Rapporteur’s first report emphasised the causes and consequences of VAW and highlighted the duty of states to address VAW. It also recommended that national governments undertake legal reforms in accordance with the model legislation that accompanied the report [43]. This report stimulated legal reforms in numerous countries, but did not immediately lead to reforms in Sri Lanka. Subsequently, at the ICES, a group of experts and VAW advocates created a Domestic Violence Task Force and developed a number of working papers to begin discussions on creating a VAW legislation in Sri Lanka [44].

International organisations based in Sri Lanka such as UNFPA also influenced and supported the response to VAW. For example, international agencies and donors were able to support NGO services for abused women, especially during the civil war and the tsunami in 2004 [16]. UNFPA and other international donors have also supported the MOH’s comprehensive VAW services and the Ministry of Women’s VAW activities [30].

#### Ideas: How was the problem of VAW framed?

The diversity of actors involved, particularly among the NGOs, inevitably leads to diverse variation in the framing of the nature of the problem of VAW, its possible solutions and policy responses.

Since the 1970’s an international feminist movement has been drawing attention to VAW as part of a global discourse of gender inequality [44]. It was not until 20 years later that VAW became a part of the larger international discourse of human rights [45]. Sri Lanka followed the international discourse at the time by identifying VAW as a human rights violation needing a response through criminal and civil law. Amendments to the Penal Code in 1995 and 1998 criminalized acts of sexual violence and aggression, though marital rape could not be prosecuted under this law [16].

While the previous section showed the general cohesion among NGO groups in shaping and supporting the PDVA Bill as a woman’s and human rights issue, there was a lack of agreement and cohesion between politicians. Although the PDVA Bill was finalised in 2001, with input from many women’s organisations [44], it was not introduced to Parliament until February 2005. There remained large disagreements on the conceptualization of the issue of partner violence and the appropriate responses to it. The women’s organizations portrayed it as a manifestation of patriarchal power and gender inequalities, and were successful in pushing most of their original draft of the PDVA Bill by framing partner violence as part of a women’s and children’s welfare agenda. Doctors also aligned with women’s NGOs and some spoke out to public meetings providing evidence regarding the number of pregnant women and children they had seen experiencing ill-health consequences of partner violence. Members of Parliament from both the then-ruling party and the opposition also endorsed the PDVA Bill, agreeing that partner violence was a human rights issue and a socially relevant problem, and that it was the responsibility of the government to acknowledge its seriousness and respond through the legal system [44]. However, many MPs and the Magistrate Court opposed the PDVA Bill, seeing IPV as part of the discourse of familial ideology and portraying partner violence as a family problem, emphasizing instead the need to protect children’s welfare. The main fear for this group was that the bill would lead to breaking up the sanctity of the family unit [44, 46]. This Government position towards marital rape, and VAW in general, is supported by conservative family values and a widespread culture of social stigma around separation and divorce, in a country where marriage is considered a sacrament [47]. In some occasions, the President, joined by several MPs and influential politicians, publicly upheld traditional family values at the expense of VAW and women’s rights [44, 48].

It was a narrow definition of partner violence as a criminal justice issue linked to the Penal Code rather than a wider health issue that prevailed in the resulting PDVA Bill, reflecting the supremacy of family ideology. This left those offences not covered in the Penal Code, such as marital rape, unrecognised as violence. The narrow definition also meant government responses were primarily focused on judicial remedies rather than on addressing health consequences and offering accessible support services to women, as originally suggested by NGOs [49].

Although the UN Human Rights Council stated in 2011 that the PDVA Bill was evidence of the Sri Lankan government’s commitment to strengthen procedural laws [49], there remain doubts amongst political leaders about the acceptability of partner violence as a legitimate policy issue within Sri Lanka. At a Women’s Day Celebration in 2010 the then President expressed feelings that the PDVA law may be weakening cultural values around the family [44]. Other political leaders also expressed their feelings that the law reflects western ideology and undermines the family unit. The 2010 national government policy of Mahinda Chinthana continued to push a family ideology that expressed women’s primary roles as child-bearing and upholding family values [41]. This framing of partner violence in Sri Lanka also aligns with the wider cultural context which tends to consider violence as a part of normal married life [44, 46]. Indeed, this attitude is reflected amongst many professionals, including police officers [50] and medical students [51]. In an exploratory mapping of organisations offering interventions services for partner violence in Sri Lanka, half of women’s organizations cited these negative social attitudes as the greatest challenge in their work to push for VAW services [39].

Internationally, in the early 2000, women’s organizations and activists in the US and Europe joined UN agencies (e.g. the World Health Organisation - WHO) in framing VAW not only as a human rights, but also as a major public health issue [52, 53]. Despite early studies documenting the pervasiveness of VAW in Sri Lanka [54], such thinking reached Sri Lanka only around 2008, with the development of a National Report on Violence and Health, jointly published with WHO [8]. This is an important milestone document for violence and health in Sri Lanka, which marks the official recognition of violence as a public health issue that needs to be tackled by the health sector. Since then, MOH policy documents have addressed violence as a key socio-health issue, as reiterated in its newly adopted *National Health Plan* [37].. Subsequently, the inclusion of the health sector as an institution responsible for implementation strategies set out by the plans to address VAW in Sri Lanka, has reinforced recognition of VAW as a public health issue and the need to strengthen the health sector response.

### Issue characteristics: What was the evidence on the health burden of VAW?

The characteristics of a policy ‘issue’ include the extent to which credible indicators or data are available to assess the size of the burden compared to other problems [14]. In order for an issue to get onto the policy agenda, it needs be recognized as a concern, often through clear measures that show the extent of the problem. Early research on VAW in Sri Lanka focused on partner and wife battering, with the first report published in 1982 [9]. Regional studies published over the past thirty years cite 20–72% of women experiencing partner violence in Sri Lanka [7], though no national prevalence data is available. Furthermore, Sri Lanka has no systematic data collection for VAW. The Committee on the Convention on the Elimination of All Forms of Discrimination against Women (CEDAW) has expressed concern for the limited availability of disaggregated data for VAW in Sri Lanka [55]. A shadow NGO report to the CEDAW Committee mentioned the unwillingness of the Ministry of Women to rely on research and professional expertise outside the ministry, despite their inability to do research independently [56]. As previously mentioned, the first ever national health-related report on VAW was the WHO Sri Lanka *National Report on Violence and Health* (2008), which highlighted the role the health sector must play in addressing VAW. Its first recommendation was to enhance capacity for collecting data on violence [8]. Meanwhile, without clearer data it remains difficult to raise the profile of VAW as a public health issue.

Even with data on the prevalence of VAW, the issue would compete with other priority health issues. For instance, Sri Lanka’s health system is currently facing high demands from an aging population and high mortality rates from non-communicable diseases [57]. Additionally, VAW does not have clear impact measures compared to other problems. The information that is available is often in the form of physical injuries experienced and reports made to police departments. Receiving information on VAW from police departments, such as the number of partner violence and sexual violence complaints filed, does not depict the health consequences nor severity of each case unless it results in a homicide. In fact, a review of research evidence on VAW in Colombo found that only 23% of abused women accessed any sort of support institution, and of those even fewer accessed police services [58].

Without credible mortality and morbidity data it is challenging to make a case for priority policy responses from the health sector, particularly in the context of the prevailing cultural attitudes about family life discussed in the previous section. However, the lack of VAW data did not stop service interventions by women’s NGOs and MOH. Besides the initial NGO response through GBV health desks in public hospitals, MOH, in collaboration with UNFPA, developed a comprehensive response to VAW in the form of One-Stop Crisis Centres (OSCCs), called Mithuru Piyasa or ‘Friendly Havens’, offering on-site medical care and basic counselling and external referral for in-depth counselling and legal aid [33, 59]. To date 33 hospitals across Sri Lanka have a Mithuru Piyasa centre [30]. A more recent model was to have medical officers (trained in mental health) offering mental health clinics for women who experienced abuse in hospitals with no on-site psychiatrists [60]. Though no evaluations were conducted to show effectiveness of such health systems responses, anecdotal evidence show that health services for VAW are still inadequate, and counselling services are minimal [61].

### Political context: What global and local structures affected a national response to VAW?

The core context issue in the Shiffman and Smith framework is global governance. The global governance structure is the degree to which norms and institutions come together at a global level to provide a platform for action on an issue, which can then influence national policies [62].

This is less of an influence nationally in Sri Lanka, nevertheless, the influence of global institutions and global policy responses is important. Sri Lanka was ravaged by a civil war that lasted nearly 3 decades (1983–2008). The United Nations and many Western states were interested in supporting Sri Lanka to move towards a peaceful resolution and enable post-conflict aid. Global influence from these international players certainly impacted the national stage [8]. The former government’s President was not fully supportive of eliminating VAW, although policy statements on gender equity were present in his election policy [63]. Within this equivocal national climate, global influences became even more important. The judicial rights-based focus of global influencers, alongside the national focus on criminalisation of violence, clearly had resonance in a post-conflict setting in which the Sri Lankan government was trying to tackle violence and promote democracy.

A globally favourable environment to VAW emerged after the Beijing Platform for Action (1995), where several international NGOs, women’s groups and governments rallied against VAW. This global climate supporting the eradication of VAW offered an opportunity for local Sri Lankan women’s groups to join forces and lobby against VAW at national level, and advocate for a new legislation (PDVA Bill). Moreover, the appointment of a Sri Lankan woman as the First Special Rapporteur on VAW also influenced the local policy context around VAW in Sri Lanka. Through her, global thinking was brought to bear on the Sri Lankan situation concerning the need for greater recognition of VAW as a policy issue and the need for a coherent national (including health) response [44].

The commitment of a national government to address VAW is often portrayed by being signatories of international treaties and ratifying international conventions. Sri Lanka has ratified many of the international conventions related to VAW, including CEDAW. Despite being signatory of these international conventions, there are still major gaps in translating them into national commitment, especially in the area of health. For example, funding for several health activities (especially on gender and reproductive health) is expected to come from the WHO and UNFPA [29]. There is no separate allocation of funds for VAW in the MOH budget, which has resulted in UN agencies supporting the majority of the capacity building activities within the health sector, even under the new National Strategic Framework. This is the other major way in which global institutions – and thinking – influences activities at a national and sub-national level. In Sri Lanka, it is best exemplified in the UNFPA’s financial and technical contribution to creating the first one-stop violence centre at the District General Hospital Matara in 2007 [33], where costs of equipment, training programmes and workshops for these activities were funded by UNFPA. There were minimal contributions by the MOH, although further provision of space, staff and infrastructure was expected to come from the ministry [33].

With political elections and regime change in 2016, a new window of opportunity seemed to have reopened for VAW to be placed again on the high-level policy agenda, where the general political atmosphere seemed freer and open to political liberalism and good governance [64]. Unlike the former regime, the newly elected government has publicly shown strong commitment to reduce VAW with the unilateral adoption of the *National Plan of Action Plan on Sexual and Gender-Based Violence* (2016) and the allocation of funds for its implementation under the budgetary framework 2017–2019 [25]. Furthermore, expansion of OSCCs to major hospitals was also mentioned in the National Health Strategic Master Plan for 2016–2025 [35].

## Discussion

The overall aim of this study was to examine past and current policy responses to VAW in Sri Lanka and, particularly, explore the role of the health sector in this response. We applied to a national level case study a policy analysis framework that was developed at a global level to understand why some critical health issues did not get onto global health policy agendas [11]. Our findings suggest that the cohesive NGO networks and influences of national and international actors in Sri Lanka, as well as how these actors frame the “idea” of VAW, were particularly important in shaping the nature of the policy response which in its early phase largely focused on criminal legislation expanding to a multi-sector response, including health, only at a later stage. Additionally, the lack of empirical data on VAW has impeded its definition and promotion as a major health issue, although the wider political context has allowed international organisations to push for and support a health response.

### Actor power and framing of ideas leading to policies

The power and influence of particular actors allows them to push their “frame” of an issue - defined as ‘“*underlying structures of belief, perception and appreciation”’ on which distinct policy positions depend*’ [65]. Sri Lanka has a strong presence of women’s organizations throughout the country, and they were critical for providing counselling services and creating political support to address VAW from an early stage. Mobilising around gender equality and women’s rights, women’s groups pushed for legal reforms on violence against women in the country, as has happened elsewhere [66]. Despite these efforts though, NGOs did not have sufficient political power (nor support from government) to shift prevailing traditional attitudes held by influential politicians vis-à-vis family roles and expectations. In addition, many NGOs did not define VAW as a “health” issue so securing a health-sector response was not their priority. The Ministry of Women was a key actor in framing VAW as a critical women’s rights violation, though its limited leadership and financial power has affected the extent to which it could influence or promote legislative recognition of VAW policies (e.g. PDVA Bill was watered down) and galvanise support for a multi-sector response to VAW that includes health. Additionally, the former government has appeared to show little political commitment to the Ministry of Women’s Affairs over the years, and particularly to the VAW cause. Without political leadership, support and financial resources, it is difficult for the national machinery for women’s issues to influence a national response on VAW. However, the renewed political will to address VAW by the newly formed government offers a window of opportunity to the Ministry of Women to gain political legitimacy to lead a concerted plan to eliminate VAW, involving 9 ministries. The Ministry of Health did not play a leading role in the fight against VAW until after 2005, when the criminalisation of partner violence – and the subsequent support from the WHO through the publication of the *National Report on Violence (2008)* – gave legitimacy to its senior policy-makers to define VAW as a public health issue and to start institutionalising a health sector response to violence. Like in other countries, the adoption of a legal framework on VAW (starting from the PDVA Act and the National Plan of Action to support the PDVA act in 2005) offered a renewed mandate for MOH to lead the services response on VAW [6, 67]. Support from international agencies to address VAW within the health sector was influential in the Sri Lankan setting, despite the unsupportive political regime at the time.

### Issue characteristics

VAW is a complex problem, difficult to define and with diverse consequences for women experiencing it. Moreover, the lack of data for policy makers on the scale and severity of the issue- in relation to mortality, morbidity and care seeking behaviour – made it difficult to profile VAW as a priority health issue requiring a multisectoral response. Furthermore, in Sri Lanka, the initial frame used for addressing VAW has been focused on human rights and gender equality, reflecting a global trend among feminist movements and international organisations and national post-civil war concerns to focus on legal response. Though important, such frame was diluted by the political and ideological contexts, which perceived partner violence as part of married life and its criminalisation a threat to family unity. It was only following a growing global recognition of economic and social rights (including the right to health), that, supported by WHO, the MOH could gather sufficient data and support to also describe VAW as a recognised public health issue, thus broadening the actors who could be involved in a more concerted response. A judicial and legal response to VAW is important but not sufficient, especially in a country like Sri Lanka, where very few women end up bringing their case to justice, and where mediation seems the proposed strategy. On the other hand, having a public health response to VAW can improve women’s access to care and reduce the stigma attached to VAW within the health sector and the wider community.

### Political context

Like other countries, international treaties and covenants have influenced and shaped the political context of the VAW movement in Sri Lanka. Internationally, the political environment has been conducive to taking action to address VAW, as norms have developed to accept it as a global human rights issue. The first Special Rapporteur on VAW played an important role in this global development. However, although the PDVA Bill was considered an indication of commitment to responding to violence, the lack of political leadership on VAW led to a slow development and implementation of wider VAW programmes and counselling services, heavily reliant on international organizations and NGOs. Despite having national policies and plans in place to increase VAW services in Sri Lanka, there is still a clear implementation gap and limited political will to join forces and support the work of NGOs. The commitment of the new government to finance a concerted multisector effort to address VAW appears promising.

### Usefulness of the conceptual framework

The Shiffman and Smith framework has been widely used to explore why issues have or have not got onto global policy agendas [68–70]. Smith and Neupane (2010) refined the framework to facilitate a better understanding of the factors that were most important in understanding the neonatal initiative in Nepal [71]. For our study, the framework also proved useful overall for analysing the different determinants that helped the issue of VAW to reach (or not reach) the national health policy agenda in Sri Lanka, though with some modifications. For Sri Lanka, the most influential factors were the power of ideas and the strength (or lack of it) of certain actors, primarily the Ministry of Women, international organisations and civil society. When reviewing the documents, it was apparent that the distinction between actor power (the strength of institutions and leaders in shaping a policy) and ideas is somewhat artificial and both are influenced and closely linked by the interactions between socio-political and contextual factors. It is thus very difficult to separate these elements when analysing policy agenda setting, as they are all closely interrelated. Therefore, in line with Smith and Neupane and social constructionist theories, we recommend to consider the nature of ideas and the level of support surrounding them, being aware of – and explicitly analysing – the interconnections between ideas, actors, power and context. Furthermore, we reinterpreted the global governance factor as the intersection of global norms and policies and national responses and actions including through individual leaders. We suggest that future studies analysing national priority generation take into account how international donor communities influence and shape national/local ideas and policy agenda setting. While the core elements in Shiffman and Smith’s framework (actor power, ideas, issue characteristics and political context) are not new and appear in multiple frameworks used in political analysis at both global and national levels, we have shown how a framework developed at a specific level (global) can be relevant for analysis at a different level (national) providing the researchers are open to the need for context-specific adaptation and address the implications and consequences of this in their analysis. The adaptations we have described here can expand the utility of the Shiffman and Smith framework for national level policy analysis.

## Conclusion

Although the health sector is recognized as an institution which needs to be involved when developing a multipronged solution, it is often a neglected player in development of legislation and there is little understanding of whether and how health sectors are involved in policy making nationally and globally on this issue. This policy analysis identified factors that have affected a health sector response to VAW in Sri Lanka. The tides are changing now with the new multisectoral plan on Sexual and Gender-Based Violence (SGBV) approved by the Cabinet, but a strong push from influential actors is still needed for an effective policy response to emerge within the health sector. High-level government officials, international agencies and MOH, all must agree to recognise VAW as a pertinent public health issue, and empirical data should be collected to support this recognition.

## Abbreviations

CEDAW: Convention on the Elimination of All Forms of Discrimination against Women
GBV: Gender-based violence
IPV: Intimate partner violence
MOH: Ministry of Health
MOW: Ministry of Women
PDVA: Prevention of Domestic Violence Act
SGBV: Sexual and Gender-based violence
